# *Pseudomonas* Inoculation Stimulates Endophytic *Azospira* Population and Induces Systemic Resistance to Bacterial Wilt

**DOI:** 10.3389/fpls.2021.738611

**Published:** 2021-09-22

**Authors:** Xian-chao Shang, Xianjie Cai, Yanan Zhou, Xiaobin Han, Cheng-Sheng Zhang, Naila Ilyas, Yiqiang Li, Yanfen Zheng

**Affiliations:** ^1^ Marine Agriculture Research Center, Tobacco Research Institute of Chinese Academy of Agricultural Sciences, Qingdao, China; ^2^ Graduate School of Chinese Academy of Agricultural Sciences, Beijing, China; ^3^ Shanghai Tobacco Group Co., Ltd., Shanghai, China; ^4^ Biological Organic Fertilizer Engineering Technology Center of China Tobacco, Zunyi Branch of Guizhou Tobacco Company, Zunyi, China

**Keywords:** soil-borne disease, rhizosphere microbiota, *Ralstonia solanacearum*, microbial keystone taxa, systemic resistance

## Abstract

Bacterial communities in the rhizosphere play an important role in sustaining plant growth and the health of diverse soils. Recent studies have demonstrated that microbial keystone taxa in the rhizosphere microbial community are extremely critical for the suppression of diseases. However, the mechanisms involved in disease suppression by keystone species remain unclear. The present study assessed the effects of three *Pseudomonas* strains, which were identified as keystone species in our previous study, on the growth performance and root-associated bacterial community of tobacco plants. A high relative abundance of *Ralstonia* was found in the non-inoculated group, while a large *Azospira* population was observed in all groups inoculated with the three *Pseudomonas* strains. Correspondingly, the activities of the defense-related enzymes and the expression levels of the defense signaling marker genes of the plant were increased after inoculation with the *Pseudomonas* strains. Moreover, the correlation analyses showed that the relative abundance of *Azospira*, the activity of superoxide dismutase, catalase, and polyphenol oxidase, and the expression of *H1N1*, *ACC Oxidase*, and *PR1 a/c* had a significantly negative (*p*<0.05) relationship with the abundance of *Ralstonia*. This further revealed that the keystone species, such as *Pseudomonas* spp., can suppress bacterial wilt disease by enhancing the systemic resistance of tobacco plants.

## Introduction

Soil is closely associated with the fitness, growth, and immunity of plants (Mendes et al., 2011; Qu et al., 2021). The balance of the soil ecosystem is key to the healthy growth of plants, whereas the outbreak of soil-borne disease is the most intuitive manifestation of an imbalance in the soil ecosystem (Berendsen et al., 2012; Rout and Southworth, 2013; Kwak et al., 2018). Many studies have shown that soil microorganisms promote plant growth through the following mechanisms: soil nutrition improvement, plant hormones modulation, and plant pathogen suppression (Rout and Southworth, 2013; Kwak et al., 2018; Mendes et al., 2018; Zhou et al., 2018). Soil nutrition improvement mainly includes inorganic phosphate solubilization and siderophore production to increase metal micronutrients, whereas plant hormones modulation largely depends on indole-3-acetic acid (IAA) production (Asea et al., 1988). The suppression of plant pathogens, which include production of catalase (CAT) enzyme, protease enzymes, ammonia gas, and siderophore, is a crucial mechanism for plants to decrease disease incidence (Chaiharn and Lumyong, 2009; Padda et al., 2017).

Studies on the rhizosphere microbiota have demonstrated that controlling soil-borne disease, by adjusting the population of beneficial microorganisms in the soil (Gardener and Weller, 2001), is the most feasible and sustainable method as it forms a biological barrier against the invasion of pathogens in the plant roots, and reduces the pathogenic bacterial colonization of the roots (Santhanam et al., 2015; Banerjee et al., 2018; Berendsen et al., 2018). Bacterial wilt is a destructive soil-borne disease caused by *Ralstonia solanacearum*, with estimated yield losses ranging from 50 to 60% and up to even 100% in the wet and mono cropping tobacco areas (Liu et al., 2016; Jiang et al., 2017). However, the control of *R. solanacearum* has been a worldwide problem due to the presence of several variants, different pathogenicity, and a wide range of hosts (Zhang et al., 2019; Wang et al., 2020). Traditional chemical pesticide not only enhances the drug resistance of pathogenic bacteria but also pollutes the environment and affects food safety (Liu et al., 2016). The positive effects of the soil microbial community on plant health have been extensively documented (Ambrosini et al., 2016; Zhang et al., 2019); also, soil microbiota has received increased attention because of its advantages, including environmental sustainability, less resistance to pathogenic bacteria, and safety to humans and livestock (Wang et al., 2020). Therefore, bacterial wilt can be effectively controlled by improving soil microbiota.

Early studies on rhizosphere microbes mainly focused on the direct inhibition effects of antagonistic microorganisms against pathogens (Raaijmakers and Weller, 1998; Alsanius et al., 2002; Bulgarelli et al., 2015). For example, rhizosphere fluorescent pseudomonads, an important bacterial genus in disease-suppressive soil, directly inhibited the growth of *Gaeumannomyces graminis* var. graminis and *Pseudomonas solanacearum* by producing the antibiotic 2,4-diacetyl phloroglucinol (Raaijmakers and Weller, 1998; Alsanius et al., 2002). In addition to direct antibiotic activity, specific beneficial microorganisms from disease-suppressive soil can also induce systemic resistance (ISR) against a broad spectrum of pathogens (Pieterse et al., 2014). Normally, certain rhizobacteria can enhance the activities of the defense-related enzymes and trigger jasmonic acid (JA)-, ethylene (ET)-, and salicylic acid (SA)-dependent elicitation of ISR in the host plants (Pieterse et al., 1998; Audenaert et al., 2002).

In our previous study, three *Pseudomonas* strains, i.e., *Pseudomonas koreensis* HCH2-3, *Pseudomonas rhodesiae* MTD4-1, and *Pseudomonas lurida* FGD5-2, were identified as keystone species of disease-suppressive soils (Zheng et al., 2021). However, the mechanisms of plant disease suppression by these keystone species are still unknown. Therefore, as an attempt to explore biocontrol agents from microbial keystone taxa associated with suppressive soil, this study was conducted to answer the following questions: (1) Do microbial keystone species (three *Pseudomonas* strains in this study) significantly promote tobacco plant growth? (2) Can the *Pseudomonas* strains reduce the abundance of *R. solanacearum* in soil? (3) How do these *Pseudomonas* strains change the rhizosphere bacterial community composition and interactions? and (4) Can these *Pseudomonas* strains ISR in tobacco plants? To achieve the above, we analyzed the plant-growth-promoting potential of the three *Pseudomonas* strains and collected root-associated microbiomes from different groups inoculated with these strains. The abundance of *R. solanacearum* was quantified using quantitative PCR (qPCR), and the rhizosphere bacterial communities were evaluated by high-throughput sequencing. Further, the activities of the defense-related enzymes and the relative expression of defense signaling marker genes against *R. solanacearum* in tobacco were quantitatively detected.

## Materials and Methods

### Bacterial Strains and Growth Condition

Three *Pseudomonas* strains, *P. koreensis* HCH2-3, *P. rhodesiae* MTD4-1, and *P. lurida* FGD5-2, were previously isolated from the rhizosphere of tobacco plants cultivated in Zunyi city, Guizhou Province, China (Zheng et al., 2021). The *Pseudomonas* strains were cultivated in Luria-Bertani (LB) medium at 28°C and stored in 20% (v/v) glycerol at −80°C.

### *In vitro* Analyses of Plant-Growth-Promoting Potential of the Bacterial Strains

#### Inorganic Phosphate Solubilization

Each *Pseudomonas* strain was estimated for its ability to solubilize a common form of inorganic phosphorus (calcium phosphate tribasic). Each strain (OD_600_=0.3, 5μl) was spot inoculated, in triplicate, onto Pikovskaya’s agar medium containing 0.5% calcium phosphate tribasic (Asea et al., 1988; Noh et al., 2021). The same volume sterile water was also inoculated as negative control. Plates were incubated at 30°C for 7days, and the inorganic phosphate solubilization was characterized by a clear halo around the bacterial colony. Importantly, the ability of the bacterial strains to solubilize inorganic phosphate (calcium phosphate tribasic) was estimated by calculating the solubilization index (SI) with following formula (Khan et al., 2015). SI = (halo + colony diameter)/colony diameter.

#### Siderophore Production

Each bacterial strain (OD_600_=0.3, 5μl) was spot inoculated, in triplicate, onto blue agar chrome azurol S (CAS) agar plates. A color change from blue to orange around the bacterial colony was expected to indicate the production of siderophore by bacteria (Kandel et al., 2017). The diameters of the orange-colored area and bacterial colony were measured after 72h of incubation at 30°C. The siderophore production index (SPI) was calculated as follows (Asea et al., 1988).

SPI = (color conversion area + colony diameter)/colony diameter.

#### Protease Activity

For evaluation of the protease enzyme activity, each *Pseudomonas* strain (OD_600_=0.3, 5μl) was spot inoculated on three Charcoal Yeast Extract agar plates, containing 1% skimmed milk powder (Chaiharn and Lumyong, 2009; Padda et al., 2017). The spotted plates were then incubated for 5days at 30°C. The clear zone surrounding the bacterial colony indicated the protease activity of the strain. The protease activity index (PAI) was calculated as follows.

PAI = (clear zone + colony diameter)/colony diameter.

#### IAA Production

For each strain, 20μl of the bacterial suspension (OD_600_=0.3) was inoculated into 20ml LB broth containing 5mm L-tryptophan. The inoculated tubes were incubated for 72h at 28°C in a shaking incubator at 150rpm in triplicate. Subsequently, 1ml of the culture supernatant (8,000×g, 15min) was mixed with 100μl of 10mm orthophosphoric acid and 2ml of the Salkowski reagent [50:30:1 ratio of distilled water, 95% (w/w) sulfuric acid, and 0.5M FeCl_3_] was added, followed by incubation for 15min at room temperature. The absorbance value was determined at the wavelength of 530nm. The amount of IAA produced by the three bacteria strains was accurately calculated by the standard curve of IAA (Supplementary Figure S1).

#### CAT Activity

To evaluate CAT production by each of the bacterial strains, a loopful of fresh bacterial culture (incubated on LB plates at 28°C for 24h) was inoculated on a sterile glass slide and then 50μl of hydrogen peroxide (3%, v/v) was added and incubated at room temperature for 1min (Puri et al., 2020). The generation of gas bubbles under the cover glass indicated positive CAT activity. This experiment was performed with three replicates for each strain.

#### Ammonia Production

To accurately determine ammonia production of each bacterial strain, 100μl of the bacterial suspension (OD_600_=0.3) was inoculated to three test tubes, each containing 10ml of peptone water (Dey et al., 2004). The tubes were incubated for 72h at 30°C, and then, 500μl of Nessler’s reagent was added into each reaction tube. Appearance of brown yellow color of liquid in test tube indicates the ammonia production of bacterial strains.

### *In vivo* Plant-Growth-Promoting Experiment of Bacteria Strains

#### Plant Materials and Growth Conditions

Tobacco plant (*Nicotiana tabacum* L.) was selected as a host plant. Seeds obtained from the Tobacco Research Institute of the Chinese Academy of Agricultural Sciences (Qingdao, China) were first grown in nursery trays using substrate for 15–20days. Well-developed rooted tobacco plants (leaves, 2–3; plant height, 3–4cm) were transferred to a plastic basin (9×6×7cm) filled with 200g of loam dry soil collected from a healthy field in Zibo city (Shandong province, China) and then placed under greenhouse conditions at 25–28°C with a relative humidity of 70% and 12h light and dark cycle.

#### Inoculation of the *Pseudomonas* Strains and Plant Growth Assessment

*P. koreensis* HCH2-3, *P. rhodesiae* MTD4-1, and *P. lurida* FGD5-2 strains were separately grown in LB liquid medium for 24h at 28°C and 150rpm. Bacteria were then collected by centrifugation (5,000×g, 10min) and resuspended in distilled water. Seven days after transplantation, 20ml of the bacterial suspension (OD_600_=0.3), or the same amount of water (negative control) was inoculated into the tobacco plant using root drenching methods (Hu et al., 2016). Bacterial inoculation was carried out weekly once for 3weeks. During the experimental period, enough water was provided to each plastic basin. Tobacco plants were grown until they showed different agronomic characters among treatments (25days after the last inoculation in this study). The investigation of agronomic traits (plant height, maximum leaf area, and numbers of effective leaves) and the collection of plant samples to determine the fresh and dry weights, and the chlorophyll-a, chlorophyll-b content in leaves were carried out immediately (Gajewska et al., 2006).

### Bacterial Community Composition

#### Experimental Design

Tobacco plants grown under the same conditions (see Section “Plant Materials and Growth Conditions”) were transferred to a plastic basin (9×6×7cm) filled with 200g of soil. Four different treatments were applied as follows: *P. koreensis* HCH2-3; *P. rhodesiae* MTD4-1; *P. lurida* FGD5-2; and only distilled water. The experiment was performed using a randomized complete block design with three replicates for each treatment, and each replicate contained six pots. Each pot was supplemented with a total of 60ml of *Pseudomonas* suspension (OD_600_=0.3, once a week for 3weeks). *R. solanacearum* RS10 (obtained from our laboratory, unpublished) was incubated in nutrient broth (NB) medium for 36h at 28°C, and the suspension (OD_600_=0.5, 20ml) was inoculated into each pot 1week after the last *Pseudomonas* treatment.

#### Rhizosphere Soil, Roots, and Leaf Sampling

Rhizosphere soil, roots, and leaf samples were collected 5days after *R. solanacearum* inoculation. All leaf samples were washed with cold distilled water, frozen with liquid nitrogen, and stored at −80°C. The sampling of the rhizosphere soil and roots was as follows: The rhizosphere soil (more than 1g per sample) which was tightly bound to tobacco roots was obtained with a brush (Zheng et al., 2021), the root was collected by rinsing thoroughly with phosphate-buffered saline (PBS) solution and then soaked in alcohol (75%, v/v) for 1min and sodium hypochlorite (5%, v/v) for 3min, and the root was finally rinsed with sterile water for three times (Zheng et al., 2021). All rhizosphere soil, roots, and leaf samples were stored at −80°C prior to DNA or RNA extraction.

#### DNA Extraction and Quantifications of *R. solanacearum* and *Nifh* Gene

Total genomic DNA was extracted from the rhizosphere soil (300mg) and roots (200mg) using the FastDNA spin kit for soil (MP Biomedicals, CA, United States), following the manufacturer’s instructions. DNA samples were dissolved in 50μl distilled water and stored at −20°C for further analysis. The DNA samples quality was evaluated based on the absorbance ratio between 260/280nm and 260/230nm using a NanoDrop2000 spectrophotometer (NanoDrop, ND2000, Thermo Scientific, DE, United States). The concentrations of the extracted DNA from all rhizosphere soils and roots were more than 20ng/μl. The abundance of *R. solanacearum* in the rhizosphere soil and roots was determined by qPCR using primers (forward: 5'-GAA CGC CAA CGG TGC GAA CT-3' and reverse: 5'-GGC GGC CTT CAG GGA GGT C-3') that targeted the *fliC* gene, which encodes the flagella subunit (Schonfeld et al., 2003). The qPCR was performed using the Applied Biosystems StepOne Plus (Applied Biosystems, CA, United States). The qPCR reaction was performed with the following conditions: initial denaturation at 95°C for 30s, 40cycles of 95°C for 5s, 60°C for 34s, and ending with melt curve analysis at 95°C for 15s, at 60°C for 1min, and at 95°C for 15s (Hu et al., 2016). Each of the 20μl reaction mixtures contained 0.8μl of each primer (10μM), 10μl SYBR Green (Thermo Fisher Scientific, Waltham, MA, United States), 0.4μl ROX (Thermo Fisher Scientific, Waltham, MA, United States), 6μl sterilized ultrapure water, and 2μl DNA sample. Standard curves were generated using 10-fold serial dilutions of a plasmid containing the 16S rRNA gene from *Arthrobacter pokkalii*, and a fragmented copy of *R. solanacearum fliC* (Zheng et al., 2021). The *nifH* gene abundance was quantified using PolF (5'-TGC GAY CCS AAR GCB GAC TC-3') and PolR (5'-ATS GCC ATC ATY TCR CCG GA-3') primer with previous reported qPCR conditions (Coelho et al., 2009).

#### PCR Amplification and Sequencing

Primers 799F/1193R (forward primer: 5'-AAC MGG ATT AGA TAC CCK G-3' and reverse primer: 5'-ACG TCA TCC CCA CCT TCC-3') were used to amplify the V5-V7 region of the 16S rRNA gene (Bulgarelli et al., 2015). PCR was performed in a 20μl mixture containing 4μl of 5×FastPfu Buffer (TransGen Biotech, China), 2μl dNTPs (2.5mm, TransGen Biotech, China), 0.8μl of each primer (5μM), 0.4μl FastPfu Polymerase (TransGen Biotech, China), 0.2μl BSA (TransGen Biotech, China), and 1μl DNA template (10ng of DNA). The qPCR reaction was performed with the following conditions: 95°C for 3min; 27cycles of 95°C for 30s; 55°C for 30s and 72°C for 45s; and 72°C for 10min. The PCR products were separated using 2% agarose gel electrophoresis, and corresponding bands were excised and purified using the MinElute PCR Purification Kit (Qiagen, Germany), and the QiagenQIAquick Gel Extraction kit (Qiagen, Germany). The purified PCR products from each sample were mixed and sequenced on the Illumina MiSeq sequencing platform at Shanghai Majorbio Bio-pharm Technology Co., Ltd., China.

#### Analysis of Sequencing Data for Bacterial Communities

16S rRNA gene sequences were processed using the Quantitative Insights Into Microbial Ecology pipeline. Based on the overlapping relationship between pair-end reads, the paired reads were merged into a sequence. Meanwhile, the quality of reads and the effect of the merge were filtered by quality control. According to the barcode and primer sequences at the beginning and end of the sequence, an effective sequence was obtained, and the sequence direction was corrected. The operational taxonomic unit (OTU) assignment was performed by Uparse (version 7.0.1090; http://drive5.com/uparse/; Edgar, 2013) at a similarity of 97%. Chao1 estimator was used to directly compare the α-diversity of the different treatment samples. principal coordinates analysis (PCoA) was applied to ordinate the microbial composition in the different treatments based on Bray-Curtis distance using the vegan and ggplot2 packages in the R software (v 3.5.3). PERMANOVA was calculated based on Bray-Curtis distance with 999 permutations using the vegan package of R. Sequence data for 16S rRNA reads have been deposited in the NCBI Sequence Read Archive under BioProject number PRJNA747861.

### Determination of the Defense-Related Enzymes in Leaves

The activities of the fundamental defense-related enzymes in tobacco leaves were evaluated to assess the systemic defenses caused by the *Pseudomonas* strains. The superoxide dismutase (SOD), peroxidase (POD), CAT, phenylalanine ammonia-lyase (PAL), and polyphenol oxidase (PPO) were extracted and measured from 0.1g of leaves using activity test kits (BC0175, BC0095, BC0205, BC0215, and BC0195, respectively, Beijing Solarbio Science & Technology Co., Ltd., China). The SOD enzyme activity in the reaction system was defined as an enzyme activity unit when the inhibition rate of the xanthine oxidase conjugate reaction system was 50%. One unit of POD and PPO activity was defined as the change of 0.005 per minute per gram sample in 1ml reaction system at A_470_ and A_410_, respectively. One CAT activity unit was defined as the degradation of 1μmol H_2_O_2_ per gram of sample per minute in the reaction system. One unit of PAL activity was defined as the change of 0.05 per minute per gram sample in a milliliter of the reaction system at A_290_. The SOD, POD, CAT, PAL, and PPO activity was expressed in U/mg protein.

### RNA Isolation and Expression Analysis of Defense Signaling Marker Genes in Tobacco by Quantitative Real-Time PCR

Total RNA was extracted from tobacco leaves, which were collected 5days after *R. solanacearum* inoculation with the Plant Total RNA Kit (ZP405, Beijing Zoman Biotechnology Co., Ltd., China), following the manufacturer’s instructions. Agarose gel electrophoresis (electrophoretic voltage 10V/cm, 25min) was used to separate and purify the total RNA. The first-strand cDNA was synthesized by HiScript III RT SuperMix for qPCR (+gDNA wiper; R323-01, Vazyme Biotech Co., Ltd., China). The corresponding primers of the defense signaling marker genes were designed as previously described (Supplementary Table S1; Tahir et al., 2017; Zhao et al., 2019; Li et al., 2020). The Quantitative Real-Time PCR was performed on the Roche LightCycler 96 Real-Time PCR System (Roche Diagnostics, Switzerland) using the ChamQ SYBR Color qPCR Master Mix (Q411-02/03, Vazyme Biotech Co., Ltd., China) under the following conditions: pre-denaturation at 95°C for 30s, followed by 40cycles of 95°C for 10s, 60°C for 30s, and dissociation curve at 95°C for 15s at 60°C for 60s at 95°C for 15s. All samples were performed in triplicate.

### Statistical Analyses

Statistical analyses were conducted by Duncan’s significance test using SPSS20.0 (IBM, Chicago, United States). Data were showed as means ± standard error (SD). Different letters in the tables and figures indicate significant difference at *p*<0.05 level.

## Results

### Plant Growth-Promoting Analyses *in vitro*

Two of the three *Pseudomonas* strains (*P. koreensis* HCH2-3 and *P. lurida* FGD5-2) were demonstrated to perform excellent inorganic phosphate solubilization, using qualitative plate tests (Supplementary Figure S2). Notably, the FGD5-2 strain solubilized the highest amounts of tri-Ca phosphate (SI, 3.97±0.15), which was significantly greater (*p*<0.05) than those by the other two strains (Table 1). Based on the diameter of the orange halo on the blue CAS agar plates (Supplementary Figure S3), all three bacterial strains showed siderophore production abilities, of which strain HCH2-3 had the largest orange halo area (Table 1). The qualitative assessment of the protease activity in the plate assays showed that all three strains were positive for protease enzyme activity (Supplementary Figure S4). The FGD5-2 and MTD4-1 strains showed a larger clearance zone on the plates than the HCH2-3 strain (Table 1). As a crucial plant growth hormone, IAA was confirmed to be produced *in vitro* by all three strains, with amounts ranging from 12 to 28μg/ml (Table 1). Among all the strains, the HCH2-3 strain produced the highest amounts of IAA. In addition, all three strains were positive for CAT enzyme activity. Particularly, the MTD4-1 strain showed the greatest activity, as observed *via* the generation of gas bubbles under cover glass (Supplementary Figure S5). All three strains produced ammonia *in vitro* (Table 1 and Supplementary Figure S6).

**Table 1:** The *in vitro* plant-growth-promoting potential of three *Pseudomonas* strains.

	Bacterial strains		
	*P. koreensis* HCH2-3	*P. lurida* FGD5-2	*P. rhodesiae* MTD4-1
Inorganic phosphate solubilization (SI)	2.15 ± 0.11^b^	3.97 ± 0.15^a^	−
Siderophore production (SPI)	2.61 ± 0.13^a^	1.89 ± 0.07^b^	2.18 ± 0.08^b^
Protease activity (PAI)	1.52 ± 0.07^b^	1.93 ± 0.09^a^	1.98 ± 0.07^a^
IAA production (μg/mL)	28.12 ± 1.09^a^	12.76 ± 1.48^c^	16.60 ± 0.25^b^
Catalase activity	+	+	+
Ammonia production	+	+	+

Values were analyzed and expressed as mean ± standard error (*n*=3) based on duplicate experiments, and data sharing the different letters were significantly different (*p*<0.05). The plus (+) means apparent activity/production; the minus (−) means no activity/production.

### *In vivo* Plant Growth-Promoting Assay of Bacterial Strains

To evaluate whether the HCH2-3, FGD5-2, and MTD4-1 strains could promote plant growth, we conducted a greenhouse experiment using tobacco seedlings. The results of *Pseudomonas* treatments illustrated the significant positive effect on the plant height, maximum leaf area, and the number of effective leaves (Table 2). All three strains significantly enhanced plant height (>60%) compared to the control group. The leaf characteristics of the tobacco plants that were inoculated with any of the three bacterial strains were significantly greater than those of the control. Only the inoculation with the HCH2-3 and FGD5-2 strains had a significant positive effect on the fresh weight of tobacco leaves, which was 21.7 and 16.5% higher than that of the control group, respectively (Table 2). However, after complete dehydration (65°C, 24–36h), the dry weight of leaves in each treatment was determined. The treatments with any of the three bacterial strains significantly increased the dry weight compared to that of the control treatment. Chlorophyll-a content in tobacco leaves increased significantly after inoculation as compared with that in the control group. Similarly, all three *Pseudomonas* strains significantly enhanced the content of chlorophyll-b as compared with that in the control. Chlorophyll content in tobacco plants after inoculation with the three bacterial strains was significantly improved, thus ensuring that plant photosynthesis ultimately promoted plant growth.

**Table 2:** Comparison of tobacco growth and morphological traits (e.g., plant height and leaf area) between the control and inoculation groups.

Plant growth parameters	Treatments			
	HCH2-3	FGD5-2	MTD4-1	Control
Plant height (cm)	5.08 ± 0.07^b^	5.83 ± 0.09^a^	4.88 ± 0.05^b^	3.05 ± 0.04^c^
Maximum leaf area (cm^2^)	38.08 ± 2.30^b^	46.40 ± 1.17^ab^	49.01 ± 2.28^a^	27.51 ± 2.17^c^
Numbers of effective leaves	4.67 ± 0.19^a^	4.67 ± 0.19^a^	5.00 ± 0.00^a^	4.00 ± 0.00^b^
Fresh weight (mg)	261.00 ± 6.24^a^	249.67 ± 2.88^a^	236.67 ± 5.97^ab^	214.33 ± 2.33^b^
Dry weight (mg)	105.67 ± 3.34^a^	96.67 ± 3.81^a^	84.00 ± 2.16^b^	48.33 ± 1.96^c^
Chlorophyll-a (mg/g FW)	1.95 ± 0.04^a^	2.02 ± 0.05^a^	1.86 ± 0.04^a^	0.91 ± 0.03^b^
Chlorophyll-b (mg/g FW)	1.01 ± 0.03^a^	0.84 ± 0.02^b^	0.75 ± 0.02^c^	0.39 ± 0.03^d^

Mean values of the growth-promoting indexes in leaves were evaluated after 25days of the last inoculation. Values were analyzed and expressed as mean ± standard error (*n=* 6), and data sharing the different letters were significantly different (*p<* 0.05). HCH2-3, *P. koreensis* HCH2-3; FGD5-2, *P. lurida* FGD5-2; and MTD4-1, *P. rhodesiae* MTD4-1.

### The Abundance of *R. solanacearum* in Tobacco Rhizosphere Soil and Root

**Figure 1:**
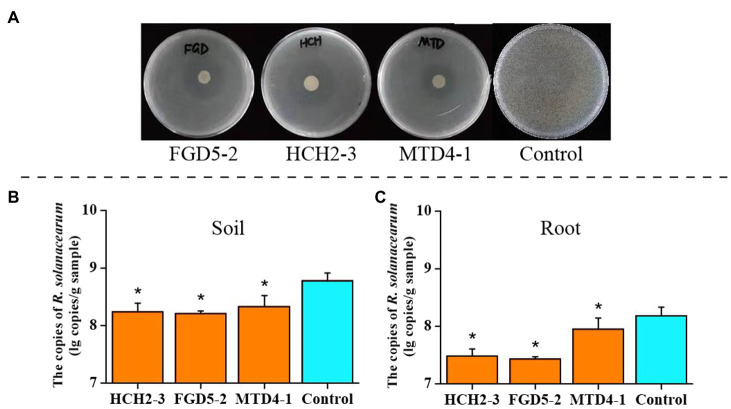
Inhibitory effect of three *Pseudomonas* strains on *R. solanacearum*. **(A)** Inhibition against *R. solanacearum* on plates. **(B)** The absolute abundance of *R. solanacearum* in soil samples. **(C)** The absolute abundance of *R. solanacearum* in root samples. Asterisks in **(B**,**C)** indicated there was significant difference between the control and inoculation groups. ^*^ *p*<0.05. HCH2-3, *P. koreensis*; FGD5-2, *P. lurida*; and MTD4-1, *P. rhodesiae*.

In addition to the plant-growth-promoting ability, the three strains could also inhibit *R. solanacearum* growth (Figure 1A). *In vivo* inoculation of *Pseudomonas* strains and the pathogen showed the significant difference (*p*<0.05, *t*-test) in the density of *R. solanacearum* between treated and non-treated (control) group by qPCR to target the flagellin gene *fliC* (Figures 1B,C). The abundance of *R. solanacearum* significantly decreased in the soil and root samples of *Pseudomonas* treated groups. Particularly, *R. solanacearum* abundance in the group inoculated with “FGD5-2” strain showed the lowest copies (7.43±0.04 lg copies of *fliC* gene per gram root). In the control group, it was 8.18±0.15 lg copies/g, which was 5.6-fold higher than “FGD5-2” treatment (Figure 1C). These results suggested that *Pseudomonas* inoculation can effectively protect their host from *R. solanacearum* infection.

### Composition of Bacterial Community

**Figure 2:**
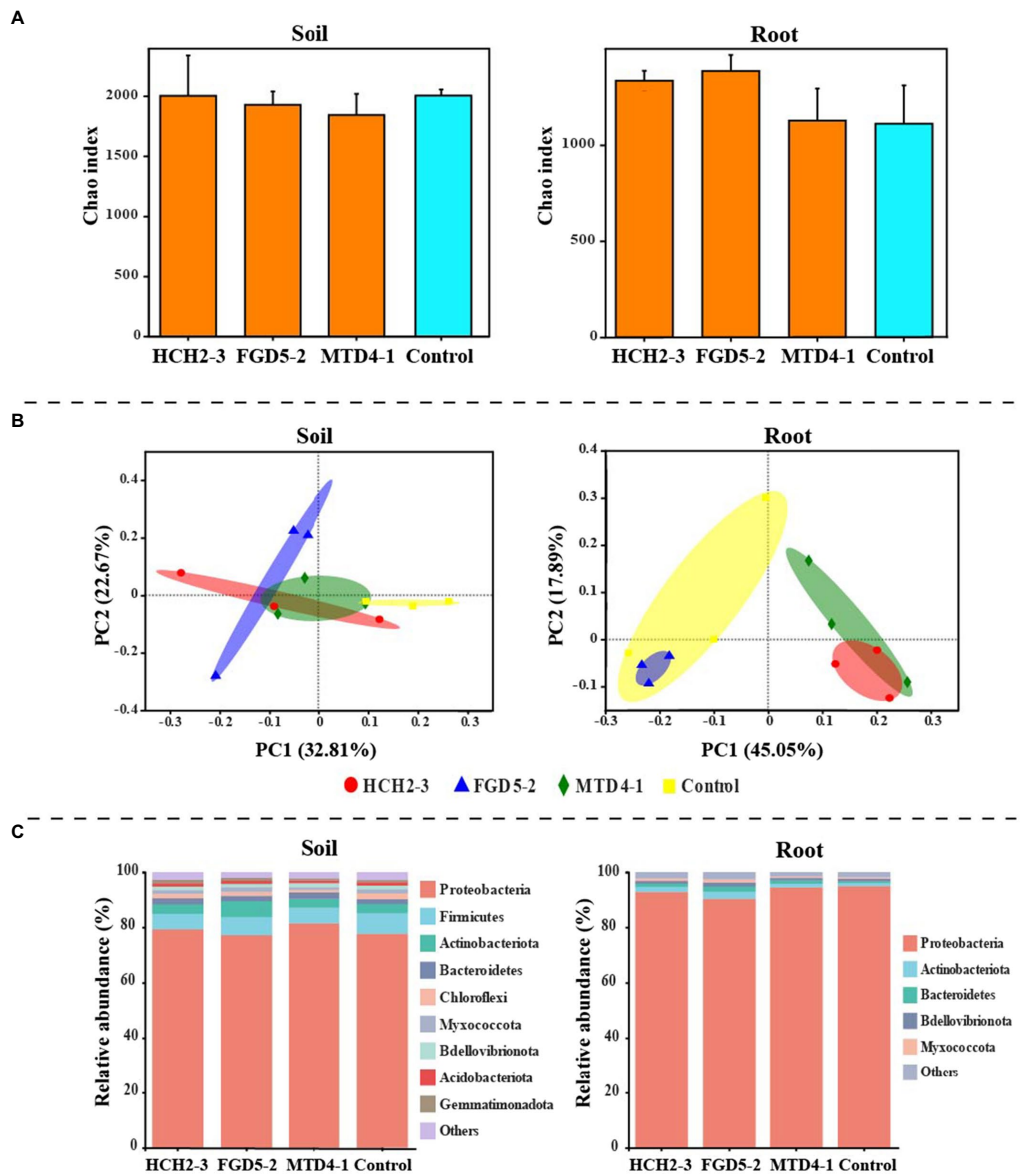
Bacterial community composition in different samples. Alpha diversity indices of bacteria in rhizosphere soil and root **(A)**. Principal coordinate analysis (PCoA) of the bacterial communities in different samples **(B)**. Bacterial community composition in soil and root samples **(C)** at the phylum level. PCoA was based on Bray-Curtis distance at the operational taxonomic unit (OTU) level. HCH2-3, *P. koreensis*; FGD5-2, *P. lurida*; and MTD4-1, *P. rhodesiae*.

A total of 1,928,721 validated sequences were produced after quality filtering. These sequences were grouped into 2,807 bacterial OTUs, and the OTU numbers ranged from 1,235 to 2,196 per sample. Compared with those of the non-inoculated group, there was no significant difference in bacterial evenness and richness indices evaluated by alpha diversity analysis (Figure 2A). However, the PCoA, based on the Bray-Curtis distance algorithm, revealed that *Pseudomonas* inoculation changed the bacterial community composition in the tobacco rhizosphere soil and root samples (Figure 2B). Consistently, permutational multivariate analysis of variance (PERMANOVA), based on the Bray-Curtis measures, showed that there were significant differences among community composition in the different groups (*R*^2^=0.64134, *p*=0.001). The relative abundance analysis indicated that Proteobacteria, Firmicutes, and Actinobacteria were dominant in the soil bacterial community, and Proteobacteria, Actinobacteria, and Bacteroidetes were the most abundant in the root bacterial community (Figure 2C). However, the relative abundance of these major phyla varied among different samples. More Proteobacteria (90.40–95.02% in root and 77.26–82.27% in soil samples) and less Actinobacteria (0.99–2.57% in root and 3.17–5.74% in soil samples) were observed in the root sample than in the soil sample. In addition, a higher relative abundance of Actinobacteria was found in the inoculated group, whereas the control group had more Firmicutes.

**Figure 3:**
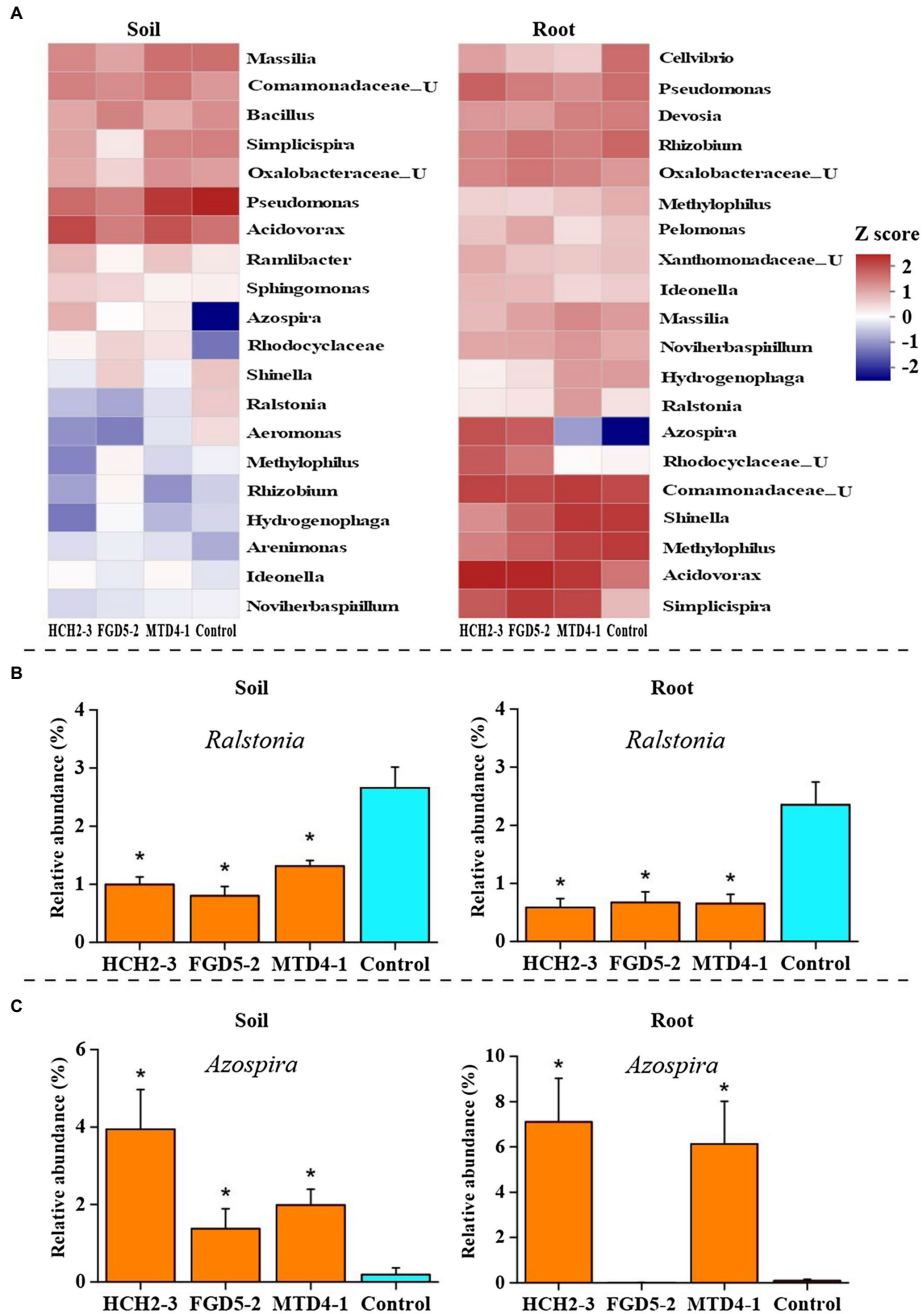
Changes in rhizosphere bacterial community composition in different samples. Bacterial community composition in soil and root samples at the genus FIGURE 3level **(A)**. The relative abundances of *Ralstonia* **(B)** and *Azospira* **(C)** in different samples. The abundance that was significantly different from control group was indicated ^*^(*p*<0.05). HCH2-3, *P. koreensis*; FGD5-2, *P. lurida*; and MTD4-1, *P. rhodesiae*.

Next, we analyzed the genus-level composition to identify genera that were significantly enriched or depleted in *Pseudomonas* treatment samples. Based on the MetaStat analysis, the relative abundances of *Azospira*, *Acidovorax,* and *Noviherbaspirillum* were higher in the inoculation groups than in the control group, while *Ralstonia*, *Shinella,* and *Methylophilus* showed the opposite trend (Figure 3A). Specifically, the relative abundance of *Ralstonia*, which was related to the occurrence of bacterial wilt, was much higher (*p*<0.05) in the rhizosphere soil (2.65%) and the roots (2.36%) of the control group than in the soil (0.80–1.31%) and roots (0.59–0.67%) of the inoculated group (Figure 3B). In contrast, compared to control group, the relative abundance of *Azospira* increased significantly (from 0.19% to 1.38–3.95% in soil samples, and from 0.09% to 6.13–7.10% in root samples) in all *Pseudomonas* treatment groups except for the FGD5-2 strain-inoculated root sample (Figure 3C). Consistently, higher *nifH* gene abundance was observed in *Pseudomonas* treatment groups (8.06–8.09 lg copies/g in soil and 8.93–9.23 lg copies/g in root) than control group (7.53 lg copies/g in soil and 8.72 copies/g in root; Supplementary Figure S7). This suggested that *Pseudomonas* can stimulate the growth of *Azospira* and *nifH* gene abundance, which might play important role in decreasing the incidence of bacterial wilt. To gain further insights into the effects of the three *Pseudomonas* strains on the bacterial community of the rhizosphere soil and root, we constructed bacterial networks based on the top 50 abundant genera that were detected in the inoculation groups. There were five genera with positive correlation and ten that negatively correlated with *Pseudomonas* (Supplementary Figure S8).

### Defense-Related Enzymes Activity

**Figure 4:**
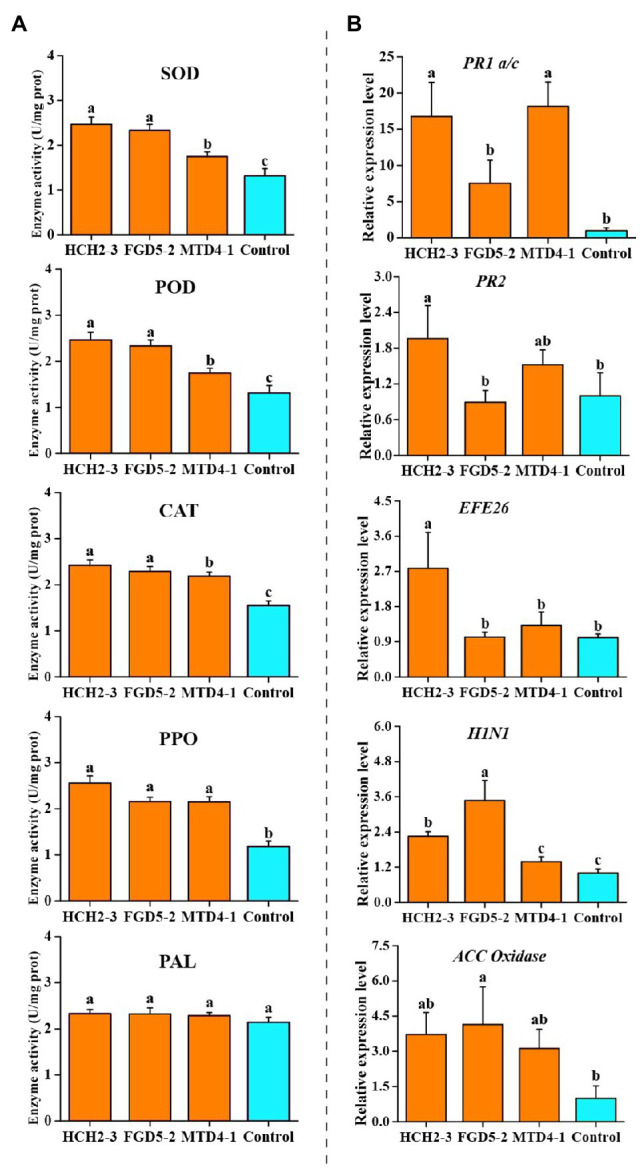
Defense-related enzymes activity **(A)** and relative expression levels of defense signaling marker genes **(B)** in the leaves of tobacco plants. Values were analyzed and expressed as mean±standard error (*n*=6) based on duplicate experiments. The different letters above the bars indicated there were significantly different (*p*<0.05). HCH2-3, *P. koreensis*; FGD5-2, *P. lurida*; and MTD4-1, *P. rhodesiae*.

To establish the activities of the defense-related enzymes of the tobacco plants after inoculating with the *Pseudomonas* strains, the SOD, POD, CAT, PPO, and PAL activities in the leaf samples were measured. Compared with those of the control group, the SOD activities of the three different treatments (HCH2-3, FGD5-2, and MTD4-1) were significantly increased (from 1.31U/mg protein to 2.46, 2.33, and 1.74U/mg protein, respectively, Figure 4A), indicating that the *Pseudomonas* strains could remove the excessive reactive oxygen species that are produced by membrane lipid peroxidation, reduce the oxidative damage of cell membrane, and improve the disease resistance of the tobacco plants. As shown in Figure 4A, the activity of POD in the group inoculated with the HCH2-3 strain (2.74U/mg protein) was significantly higher than that in other groups, while there was no significant difference in the activity among other groups. After inoculation with three single *Pseudomonas* strains, the activities of CAT in the leaf samples improved significantly. The activities of PPO in the HCH2-3, FGD5-2, and MTD4-1-inoculated samples were increased by 2.17-, 1.83-, and 1.82-fold, respectively, compared with the control group. These results showed that *Pseudomonas* inoculation can enhance the activity of multiple defense-related enzymes in tobacco leaves, which might be one of the mechanisms by which *Pseudomonas* treatment may decrease the incidence of bacterial wilt disease.

### Relative Expression of Defense Signaling Marker Genes in Tobacco Plants

To evaluate the activation of the defense signaling in tobacco after the inoculation with three the *Pseudomonas* strains, the relative expression of the defense signaling marker genes involved in SA, ET, and HR signaling in the systemic leaves was analyzed (Figure 4B; Jiao et al., 2019). Compared with the control group, HCH2-3-inoculated tobacco leaves upregulated the expression of SA signaling marker genes *PR1 a/c* and *PR2*, ET signaling marker genes *EFE26*, and HR signaling marker genes *H1N1* by 16.74-, 1.96-, 2.78-, and 2.25-fold, respectively. In addition, compared with the control group, treatment with strain FGD5-2 upregulated the expression of ET signaling marker genes, such as *ACC Oxidase* and the HR signaling marker genes *H1N1* by 4.14-and 3.47-fold, respectively. Treatment with MTD4-1 significantly activated the *PR1 a/c* and *EF1α* genes by 18.14-and 3.35-fold, respectively. The relative expressions of *PR1 a/c* in MTD4-1-inoculated samples showed the highest fold increase. Based on the above analysis, it can be concluded that at least two or more of the six defense signaling marker genes were detected in every *Pseudomonas* treated plants. This suggested that the *Pseudomonas* treatments improved the systemic resistance to *R. solanacearum* in the tobacco plants at a molecular level based on the SA, ET, and HR pathways.

### Correlations Between *Ralstonia* Abundance and *Azospira* Population, Enzymes Activity, and Signaling Marker Genes Expression

For an in-depth understanding of tobacco plant responses to pathogenic *R. solanacearum* after inoculating the keystone *Pseudomonas* strains, further correlation analyses were performed between the relative abundance of *Ralstonia* and *Azospira*, the activities of the defense-related enzymes (SOD, POD, CAT, PPO, and PAL), and the relative expression levels of the defense signaling marker genes (*PR1 a/c*, *PR2*, *EFE26*, *H1N1*, and *ACC Oxidase*) in the soil and root samples (Table 3). In the *Pseudomonas* inoculation groups, the relative abundance of *Ralstonia* was negatively correlated with SOD, CAT, *H1N1,* and *ACC Oxidase* in the soil samples. Similarly, in the root samples, there was a significant negative correlation between the abundance of *Azospira*, SOD, CAT, PPO, and *PR1 a/c,* and *Ralstonia*. One possible explanation for these results is that keystone species *Pseudomonas* helped tobacco plants resist *R. solanacearum* infection by recruiting *Azospira* population and inducing systemic resistance.

**Table 3:** Correlations between the relative abundance of *Ralstonia* and the relative abundance of *Azospira*, defense-related enzymes activity, and the relative expression levels of defense signaling marker genes in the inoculation groups.

	*Ralstonia* abundance			
	Soil		Root	
	r	*p*-value	r	*p*-value
*Azospira*	−0.517	0.089	−0.748	0.007^**^
SOD	−0.755	0.007^**^	−0.608	0.040^*^
POD	−0.315	0.318	−0.375	0.230
CAT	−0.648	0.023^*^	−0.655	0.021^*^
PPO	−0.573	0.055	−0.622	0.035^*^
PAL	−0.368	0.240	−0.371	0.235
*PR1 a/c*	−0.259	0.417	−0.587	0.049^*^
*PR2*	0.014	0.974	−0.238	0.457
*EFE26*	−0.350	0.266	−0.490	0.110
*H1N1*	−0.888	0.001^**^	−0.566	0.059
*ACC Oxidase*	−0.629	0.032^*^	−0.385	0.218

* *p<* 0.05;

** *p<* 0.01.

## Discussion

Bacterial wilt is a soil-borne bacterial disease, which can cause a devastating loss to the tobacco industry. Therefore, it will be meaningful to develop an efficient method to control tobacco bacterial wilt absolutely (Liu et al., 2016; Wang et al., 2020). There is evidence that specific beneficial microbes can increase plant biomass accumulation, productivity, and reduce soil-borne disease (Zheng et al., 2021). Based on our previous study, *Pseudomonas* species showed high degree level in the microbial co-occurrence network of disease-suppressive soil and identified as keystone taxa. Furthermore, the results of the greenhouse experiments showed that three representative strains (i.e., *P. koreensis* HCH2-3, *P. rhodesiae* MTD4-1, and *P. lurida* FGD5-2) could significantly reduce disease indices of tobacco plants. Therefore, these keystone species have the potential to act as biocontrol agents to control bacterial wilt disease for tobacco. Here, we tested how such keystone taxa contributed to disease control and plant growth promotion.

The solubilization of inorganic phosphate, as an important nutrient acquisition trait of beneficial microorganisms, we first tested this activity of the three *Pseudomonas* strains. We observed that strains HCH2-3 and FGD5-2 could solubilize tri-Ca phosphate in the plate assays (Supplementary Figure S2), which demonstrated that strain HCH2-3 and FGD5-2 could provide steady soluble phosphorus resources for the normal growth and development of the plants. Though strain MTD 4–1 cannot solubilize inorganic phosphate, it harbors other potential plant-growth-promoting abilities (see below). Siderophores play an important role in plant soil-borne disease protection by scavenging and sequestering iron from phytopathogens, and inducing systemic resistance in plants (van Loon et al., 2008; Saha et al., 2016). We found that the three *Pseudomonas* strains had the ability to produce siderophores (Supplementary Figure S3). Three *Pseudomonas* strains could convert azocasein to tyrosine using protease enzyme (Supplementary Figure S4) that is linked to the ability to effectively colonize the plant roots (Hurek et al., 1994; Puri et al., 2020). In this study, we also observed that all three *Pseudomonas* strains were able to synthesize IAA and produce ammonia and CAT enzyme (Table 1), which are beneficial to plant growth and disease resistance (Kamilova et al., 2006; Latha et al., 2009; Bumunang and Babalola, 2014).

The changes of plant associated microbial community structures, abundance, and diversity might lead to disease outbreak (Berendsen et al., 2018; Zheng et al., 2021). Adjusting the composition of the rhizosphere bacterial community is a novel strategy that has been developed to resist *R. solanacearum* infection (Wei et al., 2018). The results of our study demonstrated that *Pseudomonas* inoculation changed the bacterial community composition in the tobacco rhizosphere soil and root samples. Especially, we observed that the relative abundance of *Azospira* was far higher in the *Pseudomonas* inoculated group than in the control group (Figure 3C). The *Azospira* genus is an important nitrogen-fixing bacteria, belonging to the class of Betaproteobacteria, and has been reported to promote plant growth and resist diseases (Bae et al., 2007). Consistently, the higher copy number of *nifH* gene was observed in the *Pseudomonas* inoculated group than in the control group, which further suggested that the potential beneficial effect of *Pseudomonas* (Supplementary Figure S7). In contrast, the *Ralstonia* genus related to pathogenic *R. solanacearum* showed lower relative abundances in the inoculation groups than in the control group (Figure 3C), which was in line with qPCR result that showing lower density of *R. solanacearum* in the inoculation groups.

Previous studies demonstrated that microorganisms can enhance the resistance of plants to pathogens by activating the activity of the defense-related enzymes and the expression of defense signaling marker genes (Govind et al., 2016; Tahir et al., 2017; Jiao et al., 2019). In this study, our data showed that the activities of SOD, CAT, and PPO in the leaves were improved significantly after the inoculation with three *Pseudomonas* strains (Figure 4A). SOD is the most important enzyme in plant’s self-defense system, and it can transform O_2_-with stronger toxicity into an H_2_O_2_ with weaker toxicity (Ju et al., 2014). CAT can remove peroxide (H_2_O_2_) and cooperate with SOD to reduce the damage of active oxygen and free radicals in the membrane system (Peng et al., 2019). PPO plays a role in phenol metabolism which is related to the synthesis of lignin and quinone compounds to protect plants under disease stress (Ju et al., 2014). Similarly, the relative expression of the defense signaling marker genes that are involved in SA, ET, and HR-pathways was analyzed in the tobacco plants. A previous study showed that the tobacco plants acquired systemic resistance mainly *via* the SA-pathway (Govind et al., 2016). However, we found the relative expression of SA signaling marker genes *PR1 a/c* and *PR2*, the ET signaling marker genes *EFE26* and *ACC Oxidase*, and the HR signaling marker genes *H1N1* increased in the *Pseudomonas* treated groups (Figure 4B). This suggested that SA, ET, and HR pathways played important roles in enhancing the bacterial wilt disease resistance of tobacco plant.

## Conclusion

Three *Pseudomonas* strain, identified as keystone species in our previous study, showed excellent plant-growth-promoting potential *in vitro*, including the inorganic phosphate solubilization, siderophore production, IAA production, protease and CAT activity, and ammonia production assays. The greenhouse experiments further showed that these *Pseudomonas* strains can promote the growth of tobacco plants. Moreover, we found that *Pseudomonas* inoculation changed the root-associated microbial communities by stimulating the *Azospira* population and decreasing the relative abundance of *R. solanacearum* in the soil and root samples. *Pseudomonas* inoculation also improved the activity of the specific defense-related enzymes and activated the defense signaling marker genes of the tobacco plants, which probably induced the systemic resistance to bacterial wilt disease.

## Data Availability Statement

The original contributions presented in the study are publicly available. This data can be found at: https://www.ncbi.nlm.nih.gov/bioproject/PRJNA747861.

## Author Contributions

YZ, XC, and YL designed the experiments. X-cS, NI, and YZ performed the laboratory measurements. YZ, X-cS, and C-SZ analyzed the data and created the graphs. XH provided the critical suggestions. YZ and X-cS wrote the paper. All authors discussed the results and approved the final version of the manuscript.

## Funding

This research was founded by the Science and Technology Project of Guizhou Tobacco Corporation (201809), and the Agricultural Science and Technology Innovation Program of China (ASTIP-TRIC07).

## Conflict of Interest

XC was employed by Shanghai Tobacco Group Co., Ltd. XH was employed by Zunyi Branch of Guizhou Tobacco Company.

The remaining authors declare that the research was conducted in the absence of any commercial or financial relationships that could be construed as a potential conflict of interest.

## Publisher’s Note

All claims expressed in this article are solely those of the authors and do not necessarily represent those of their affiliated organizations, or those of the publisher, the editors and the reviewers. Any product that may be evaluated in this article, or claim that may be made by its manufacturer, is not guaranteed or endorsed by the publisher.
