# Supplementary material for: Pseudomonas Inoculation Stimulates Endophytic Azospira Population and Induces Systemic Resistance to Bacterial Wilt
Source: Front Plant Sci. 2021 Sep 22;12:738611. doi: 10.3389/fpls.2021.738611 (PMC9673043; doi:10.3389/fpls.2021.738611)
Supplement: Supplementary file 1 [file Data_Sheet_1.doc]

**Supplementary Information**

***Pseudomonas* inoculation** **stimulates** **endophytic *Azospira* population and induces systemic resistance to bacterial wilt**

**Xian-chao Shang^a,b 1^, Xianjie Cai^c 1^, Yanan Zhou^a^, Xiaobin Han^d^, Cheng-Sheng Zhang^a^, Naila llyas^a,b^, Yiqiang Li^a,*^, Yanfen Zheng^a,*^**

^a^ Marine Agriculture Research Center, Tobacco Research Institute of Chinese Academy of Agricultural Sciences, Qingdao, 266101, China

^b^ Graduate School of Chinese Academy of Agricultural Sciences, Beijing 100081, China

^c^ Shanghai Tobacco Group Co., Ltd., Shanghai 200082, China

^d^ Biological Organic Fertilizer Engineering Technology Center of China Tobacco, Zunyi Branch of Guizhou Tobacco Company, Zunyi, 563000, China


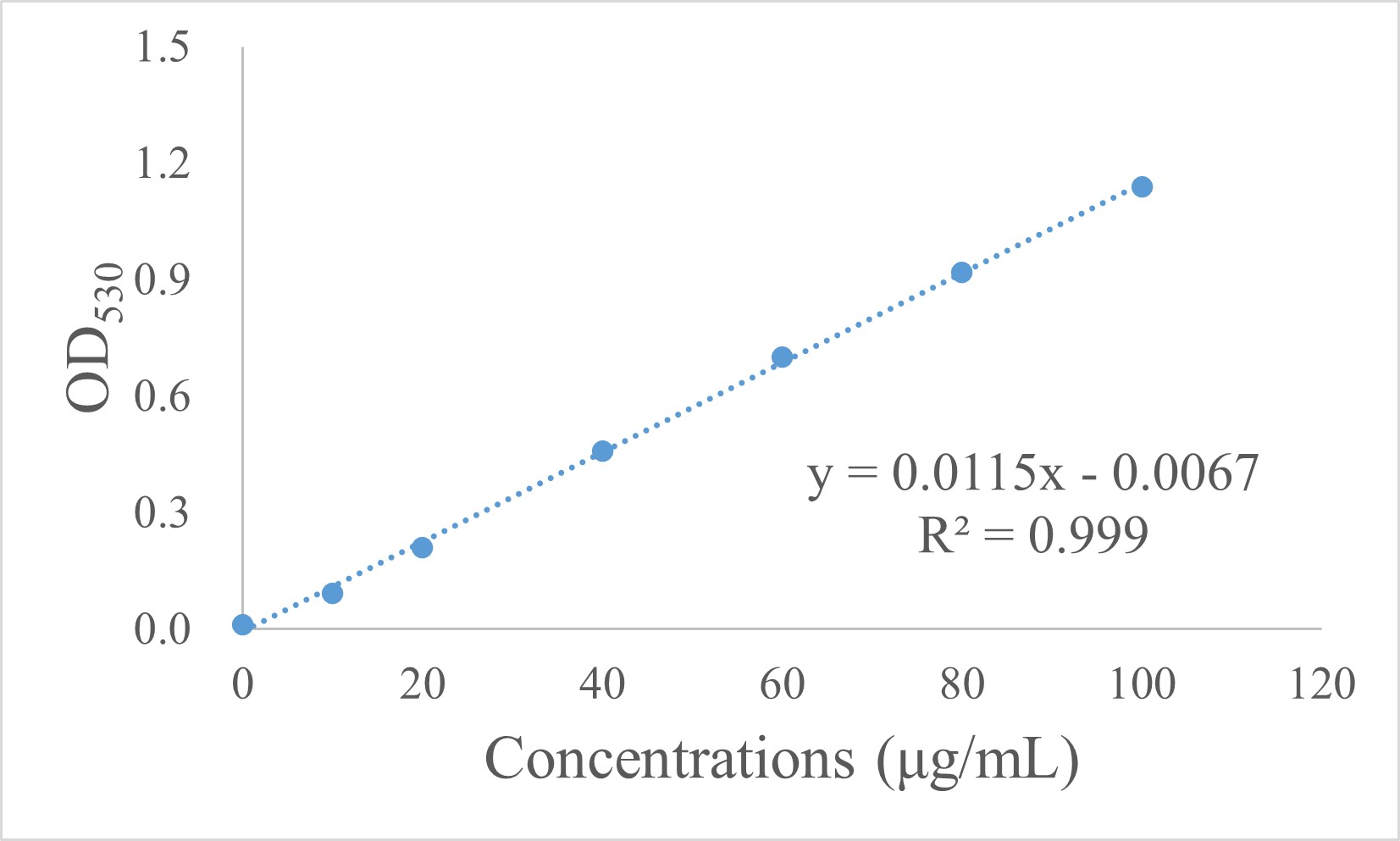


**Figure S1. The standard curve of IAA.**


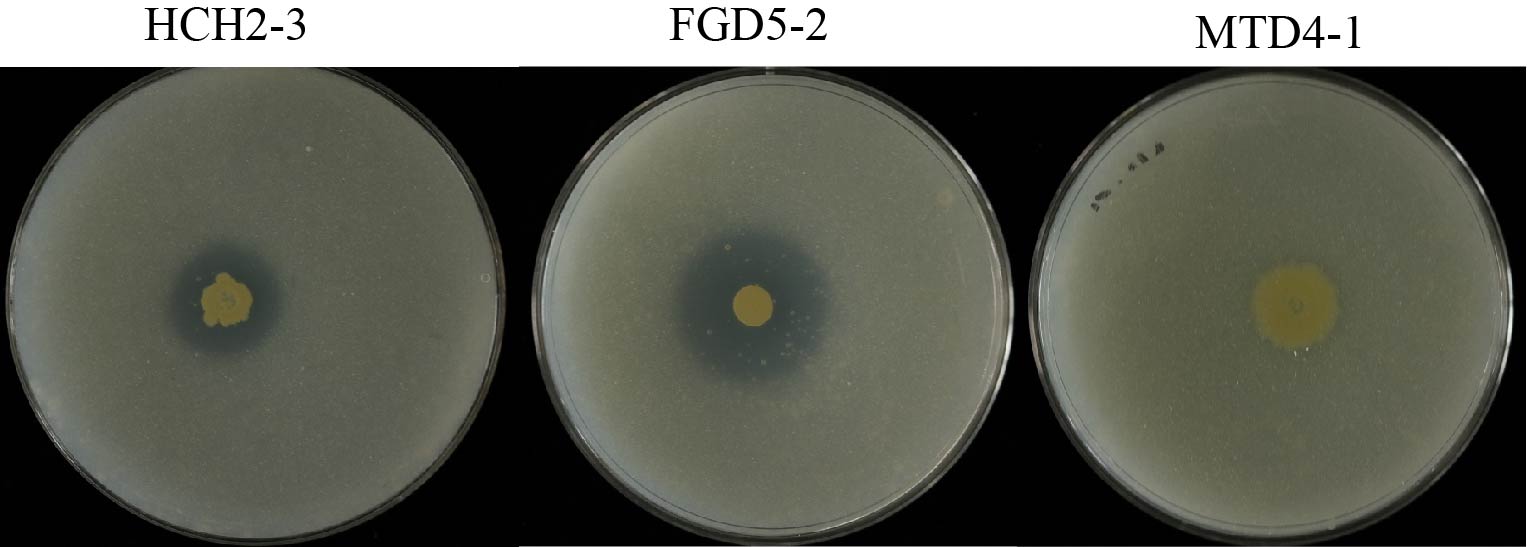


**Figure S2. The inorganic phosphate solubilization of three bacterial strains.** HCH2-3, *P. koreensis*; FGD5-2, *P. lurida*; MTD4-1, *P. rhodesiae*.


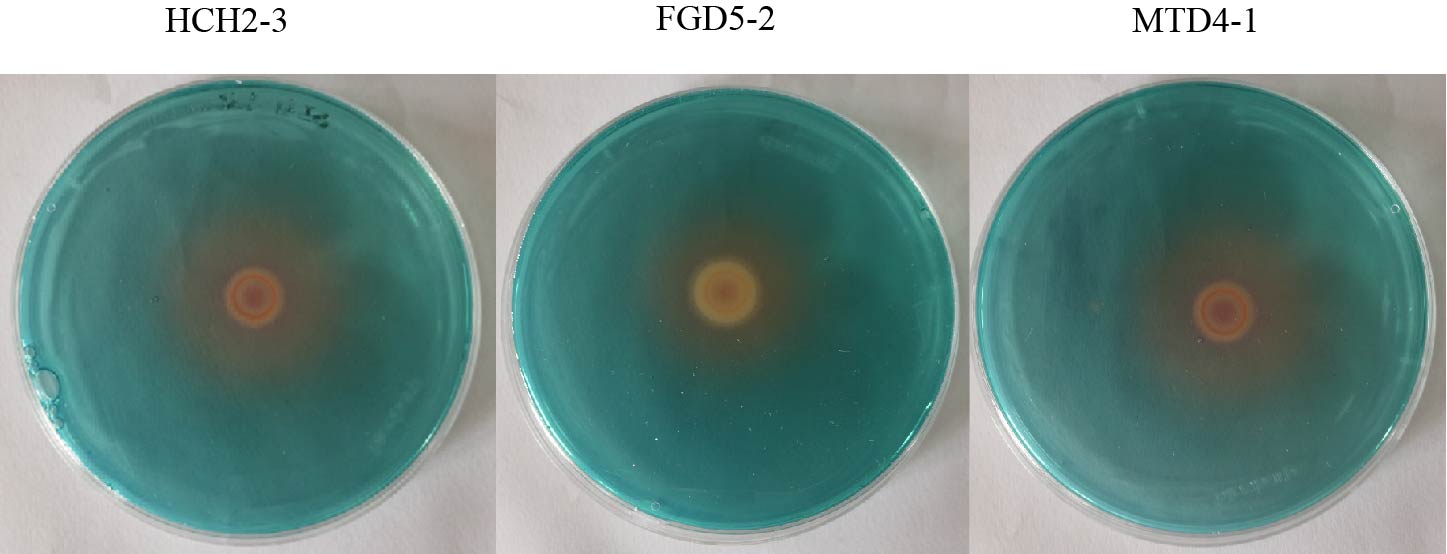


**Figure S3. The siderophore production of three bacterial strains.** HCH2-3, *P. koreensis*; FGD5-2, *P. lurida*; MTD4-1, *P. rhodesiae*.


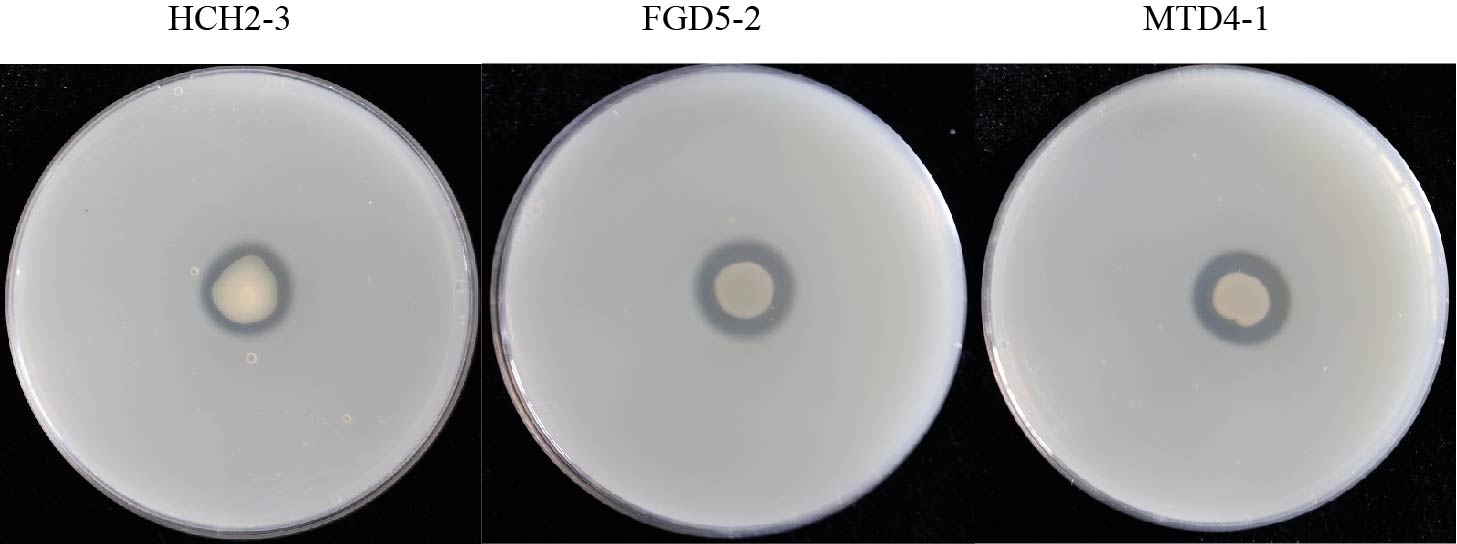


**Figure S4. The protease enzyme activity of three bacterial strains.** HCH2-3, *P. koreensis*; FGD5-2, *P. lurida*; MTD4-1, *P. rhodesiae*.


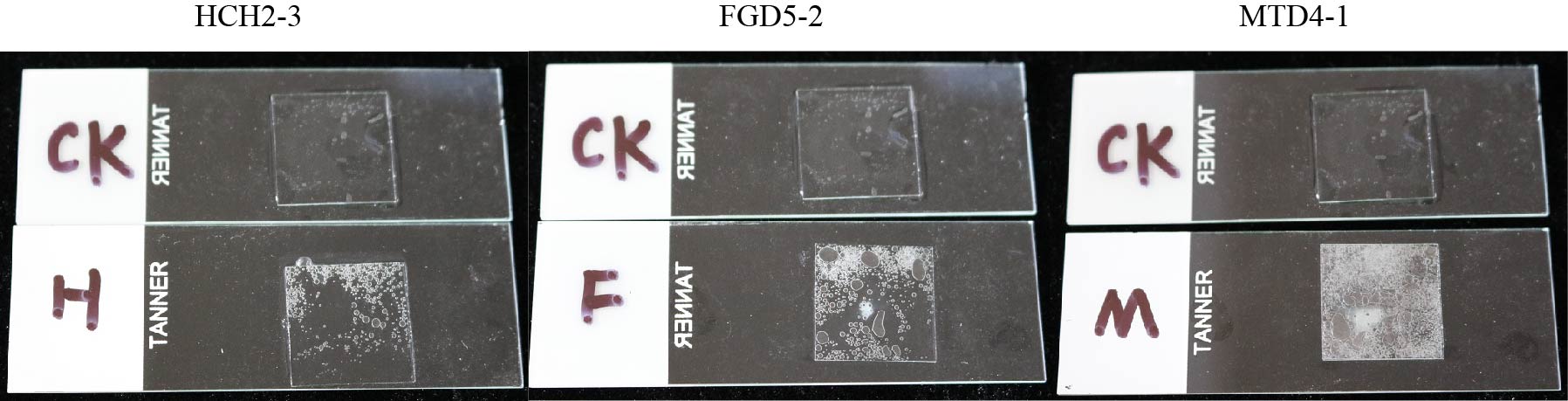


**Figure S5. The catalase enzyme activity of three bacterial strains.** HCH2-3, *P. koreensis*; FGD5-2, *P. lurida*; MTD4-1, *P. rhodesiae*.


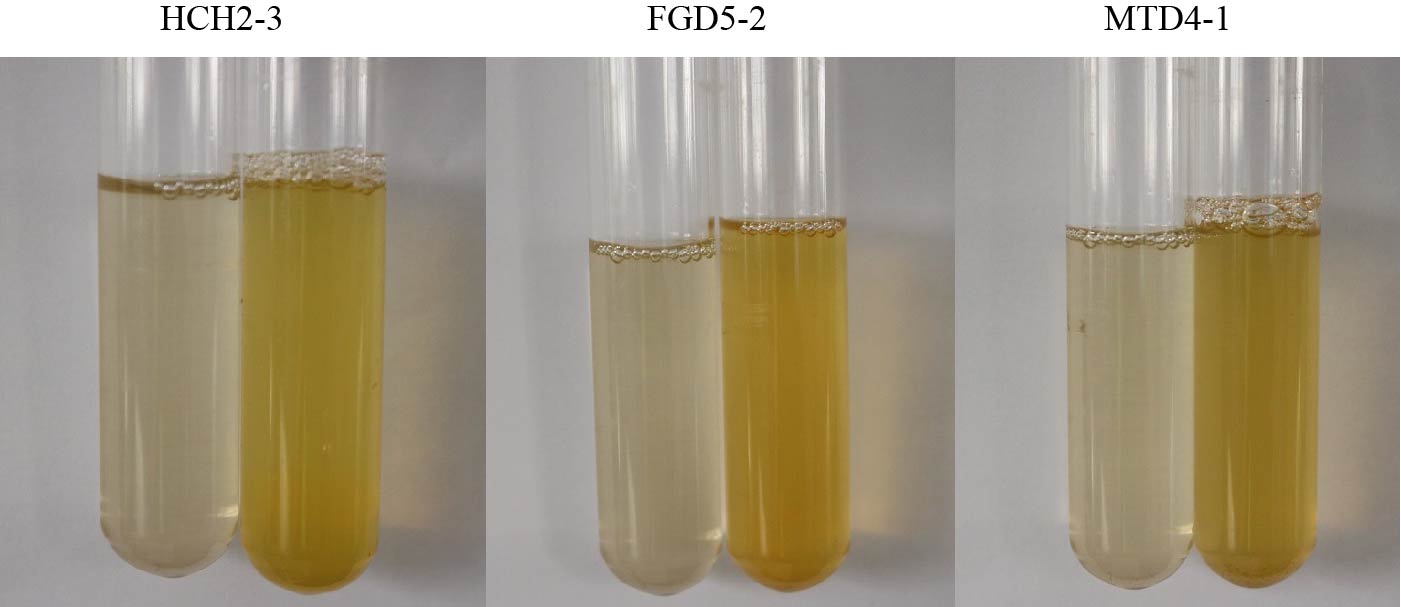


**Figure S6. The production of ammonia in vitro of three bacterial strains.** HCH2-3, *P. koreensis*; FGD5-2, *P. lurida*; MTD4-1, *P. rhodesiae*.

**
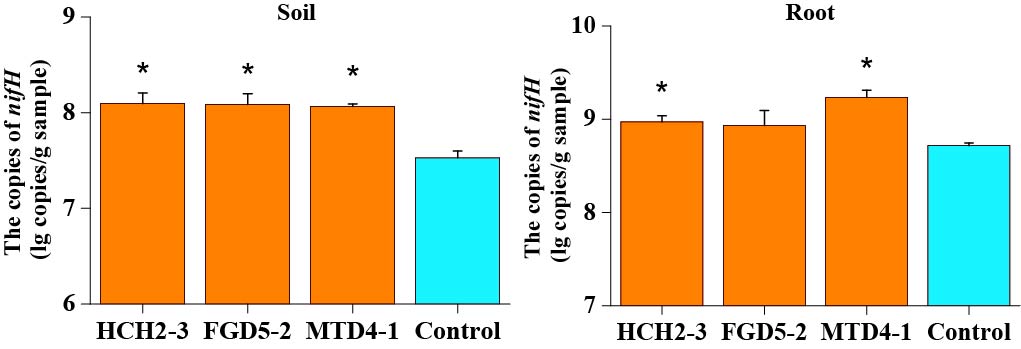
**

**Figure S7. The absolute abundance of nifH gene in soil and root samples.** Asterisks indicated there was significant difference between the control and inoculation groups. **P* < 0.05. HCH2-3, *P. koreensis*; FGD5-2, *P. lurida*; MTD4-1, *P. rhodesiae*.


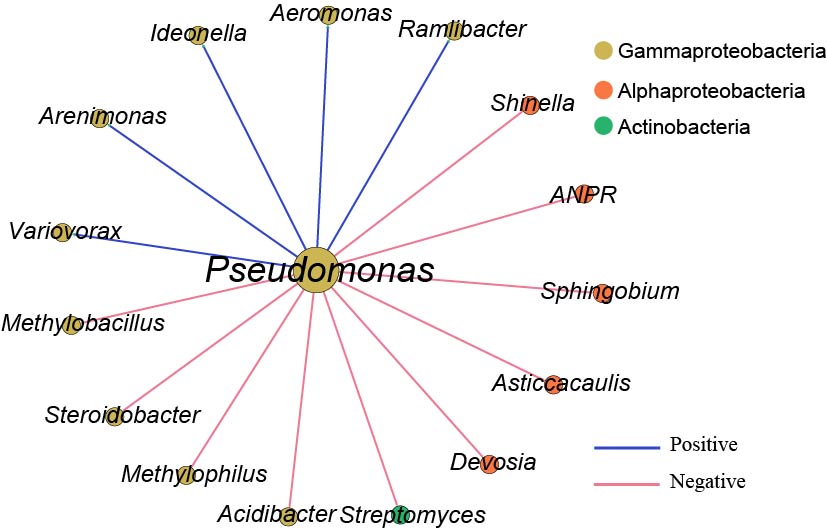


**Figure S8. Co-occurrence network analyses of the rhizosphere soil and root based on top 50 abundant bacterial genera.**

**Table S1. Primers used in this study**

| Gene | Forward primer | Reverse primer |
| --- | --- | --- |
| *PR1 a/c* | AACCTTTGACCTGGGACGAC | GCACATCCAACACGAACCGA |
| *PR2* | TGATGCCCTTTTGGATTCTATG | AGTTCCTGCCCCGCTTT |
| *EFE26* | CGGACGCTGGTGGCATAAT | CAACAAGAGCTGGTGCTGGATA |
| *ACC Oxidase* | GACAAAGGGACATTACAAGAAGT | GAGAAGGATTATGCCACCAG |
| *H1N1* | CGACCTAACAAAGTCAAGTTCTACG | CTCTATCTCCCAATAAAACCAAGC |
| *EF1α* | TGCTGCTGTAACAAGATGGATGC | GAGATGGGGACAAAGGGGATT |
